# Confinement, partial sleep deprivation and defined physical activity–influence on cardiorespiratory regulation and capacity

**DOI:** 10.1007/s00421-021-04719-z

**Published:** 2021-06-02

**Authors:** Jessica Koschate, Uwe Drescher, Uwe Hoffmann

**Affiliations:** 1 Geriatric Medicine, Department for Health Services Research, School of Medicine and Health Sciences, Carl Von Ossietzky University Oldenburg, Ammerländer Heerstr. 140, 26129 Oldenburg, Germany; 2 German Sport University Cologne, Am Sportpark Müngersdorf 6, 50933 Cologne, Germany; 3 Institute of Exercise Training and Sport Informatics, Exercise Physiology, German Sport University Cologne, Am Sportpark Müngersdorf 6, 50933 Cologne, Germany

**Keywords:** Confinement, Heart rate regulation, Exercise training, Sleep restriction

## Abstract

**Introduction:**

Adequate cardiorespiratory fitness is of utmost importance during spaceflight and should be assessable via moderate work rate intensities, e.g., using kinetics parameters. The combination of restricted sleep, and defined physical exercise during a 45-day simulated space mission is expected to slow heart rate (HR) kinetics without changes in oxygen uptake (${\dot{\text{V}}\text{O}}_{{2}}$) kinetics.

**Methods:**

Overall, 14 crew members (9 males, 5 females, 37 ± 7 yrs, 23.4 ± 3.5 kg m^−2^) simulated a 45-d-mission to an asteroid. During the mission, the sleep schedule included 5 nights of 5 h and 2 nights of 8 h sleep. The crew members were tested on a cycle ergometer, using pseudo-random binary sequences, changing between 30 and 80 W on day 8 before (MD-8), day 22 (MD22) and 42 (MD42) after the beginning and day 4 (MD + 4) following the end of the mission. Kinetics information was assessed using the maxima of cross-correlation functions (CCF_max_). Higher CCF_max_ indicates faster responses.

**Results:**

CCF_max_(HR) was significantly (*p* = 0.008) slower at MD-8 (0.30 ± 0.06) compared with MD22 (0.36 ± 0.06), MD42 (0.38 ± 0.06) and MD + 4 (0.35 ± 0.06). Mean HR values during the different work rate steps were higher at MD-8 and MD + 4 compared to MD22 and MD42 (*p* < 0.001).

**Discussion:**

The physical training during the mission accelerated HR kinetics, but had no impact on mean HR values post mission. Thus, HR kinetics seem to be sensitive to changes in cardiorespiratory fitness and may be a valuable parameter to monitor fitness. Kinetics and capacities adapt independently in response to confinement in combination with defined physical activity and sleep.

## Introduction

One of the main goals of life sciences in space research is to maintain the physical and cognitive fitness of individuals for future long-duration travels to the moon, Mars or even beyond (Strangman et al. 2014; Fomina et al. 2017; Norsk 2020). Safe, and time efficient diagnostic tools are needed to assess physical fitness in conditions without the possibility of medical interventions or close surveillance. Peak and/or maximal exercise performance [e.g., peak oxygen uptake capacity (${\dot{\text{V}}\text{O}}_{{{\text{2peak}}}}$); maximal oxygen uptake (${\dot{\text{V}}\text{O}}_{{{\text{2max}}}}$)] (Hawkins et al. 2007; Poole and Jones 2017) as well as ventilatory thresholds (VTs) (Poole and Jones 2017) are usually assessed applying cardiopulmonary exercise tests and provide a comprehensive description of individual aerobic capacity and physical fitness. Another possibility to describe the functionality of the cardiorespiratory system is to analyze the response times (kinetics) of the different physiological parameters, e.g. heart rate (HR) and oxygen uptake (${\dot{\text{V}}\text{O}}_{{2}}$) after changes in work rate (WR). The analysis of kinetics of the cardiorespiratory system offers valuable insights in cardiovascular and metabolic regulation (Hughson et al. 2001; Grassi 2000,2006; Rossiter 2011) and can be performed applying moderate WR intensities. One possibility to evaluate cardiorespiratory kinetics are WR protocols with sequences of pseudo randomly changing intensities (Hoffmann et al. 2013; Drescher et al. 2015; Koschate et al. 2016b). So-called pseudo random binary sequences (PRBS), as used by e.g., Hoffmann et al. (2013), are comparable to metabolic demands during every day habitual activities (Rossiter 2011). Since the assessment does not require high motivation or close medical surveillance, as would be the case for the assessment of ${\dot{\text{V}}\text{O}}_{{{\text{2peak}}}}$ (Myers et al. 2014), the analysis of cardiorespiratory kinetics might be a valuable approach to monitor fitness during long duration missions to space, while supervision by medical staff is not directly available.

After prolonged missions to the International Space Station (ISS), a slowing of muscular oxygen uptake (${\dot{\text{V}}\text{O}}_{{{\text{2musc}}}}$) kinetics was observed (Hoffmann et al. 2016). Additionally, the change in HR kinetics from pre- to post-flight was associated with changes in ${\dot{\text{V}}\text{O}}_{{{\text{2peak}}}}$. These decrements in physical performance during space flight are caused by the combination of microgravity, sleep problems (Barger et al. 2014; Yi et al. 2015; Mairesse et al. 2019), high and very controlled daily workloads as well as the confined living situation (De La Torre et al. 2012). Except for microgravity, these conditions can be simulated at the Lyndon B. Johnson Space Center of the National Aeronautics and Space Administration (NASA) in Houston, inside the Human exploration research analog (HERA) facility.

In former space analog conditions, using confined habitats, greater parasympathetic system activity during the wake time in the resting state was assessed throughout simulated space missions of 125 and 520 d (Vigo et al. 2012, 2013). After 70 d of confinement in a submarine, no changes in ${\dot{\text{V}}\text{O}}_{{{\text{2peak}}}}$, but an increase in the VT was reported in an exercise group, while decrements for the control group were observed in both parameters (Bennett et al. 1985).

Results from (partial) sleep deprivation experiments indicate a correlation between the onset of fatigue and heart rate variability (HRV) (Fogt et al. 2011), as well as higher sympathetic system activity, elevated autonomic stress and reduced HRV (Dettoni et al. 2012; Glos et al. 2014; Fogt et al. 2011; Liu et al. 2015), whereas after a fatiguing physical activity program, higher parasympathetic system activity was described (Jouanin et al. 2004). Greater global HRV in turn was associated with higher energy expenditure in everyday life and better perceived health status (Buchheit et al. 2005). HR kinetics seem to be very individual and react to the different physical and psychological stresses, the person experiences (Ludwig et al. 2018). To date, no information on HR kinetics, assessed during physical exercise as another indicator of neural control of HR, after sleep deprivation or during confinement is available. Since physical activity is restricted in a confined space, HR kinetics could be slowed, but if parasympathetic system activity is increased (Vigo et al. 2012, 2013), HR kinetics should be faster during isolation (Coote 2010).

The analysis of kinetics parameters during exercise can potentially help to monitor individual physical fitness during the isolation phase and may provide a tool for the estimation of performance in daily activities, which require an appropriate level of physical fitness.

On the basis of previous work in this field, the following hypotheses were investigated: the combination of restricted sleep and physical activity during 45 days of confinement (i) does not affect peak exercise capacity and ${\dot{\text{V}}\text{O}}_{{{\text{2musc}}}}$ kinetics, but (ii) HR kinetics are slowed as a result of partial sleep deprivation in combination with the limited possibility to be physically active in the confined habitat.

## Methods

### Participants

The following inclusion criteria were applied: age range from 26 to 60 years, technical skills demonstrated through professional experience, motivation and work ethics similar to the current astronaut population, psychological qualification, at least bachelor’s degree in engineering, biological science, physical science or mathematics. Ethical approval was obtained from the Institutional Review Board at the NASA Johnson Space Center (Protocol number: Pro2320) as well as the Ethical Committee of the German Sport University Cologne (Protocol number: 074/2016), and the experiments were conducted in accordance with the Declaration of Helsinki (including its amendments until 2013). Written informed consent was available from all participants prior to the experiments.

Overall, the HERA campaign four consisted of five 45 d missions, including four crew members each (*N* = 20). Due to Hurricane Harvey in August 2017, one mission with four participants had to be terminated early, and it was not possible to analyze these data sets. Further, data of two subjects could not be included for data analysis, because they had to terminate one of the exercise tests during the mission early. Both individuals exceeded the allowed HR or blood pressure values during the moderate exercise test protocol set by NASA medical board. Therefore, data of 14 individuals (9 males, 5 females) were included for statistical analyses. Anthropometric data of the subjects are shown in Table 1.

**Table 1:** Anthropometric data of the crew

*N* = 14	MD-8		MD22		MD42		MD + 4	
	Mean	SD	Mean	SD	Mean	SD	Mean	SD
Age (years)	37	7						
Height (cm)	176	9						
Body mass (kg)	73.4	14.6	72.3	13.8	72.3	13.8	72.3	13.5
BMI (kg m^2^)	23.4	3.5	23.3	2.9	23.1	3.0	23.1	3.0

*MD-8* 8 days before the beginning of the mission, *MD22* mission day 22, *MD42* mission day 42, *MD + 4* 4 days after the end of the mission

### Study outline

The five 45-d-mission scenarios were identical and designed as follows: baseline data collection was performed over the two weeks prior to the isolation period. During the mission, the individuals simulated a mission to the asteroid ‘Geographos’ and back. Five consecutive nights per week they were restricted to five hours of sleep per night and during the weekends, two nights of eight hours of sleep were scheduled. During the missions three to five, an experimental lighting protocol was tested. One hour prior to sleep, the light was blue-depleted and two hours post sleep, the light was blue-enriched. Since only one mission (*n* = 4) was finished using the standard lighting protocol, the lighting procedures will not be included in the statistical analyses.

Every second day, the crew exercised either on a cycle ergometer or they performed toning and stretching exercises over a time period of 30 min, while the allowed HR during exercise was restricted to below 85% of their age adjusted maximum.

The subjects filled in a questionnaire about the general frequency of their exercising habits and their physical activities during the year before the first exercise test prior to the beginning of the mission.

### Exercise test protocol

Before, during and after the mission, a standardized exercise test was applied to determine the kinetics responses in the moderate exercise range. The tests were scheduled eight days before the start of the mission (MD-8), on day 22 (MD22) and day 42 (MD42) of the mission, as well as four days after the mission ended (MD + 4). Eight hours ahead of the exercise test, the subjects stopped to consume alcohol and nicotine eight hours before, they stopped drinking caffeine four hours prior to the test and they started fasting two hours ahead of the beginning of the test. Additionally, eight hours before the test, no exercise was permitted and 24 h prior to the test session no maximal exercise was allowed. The exercise tests on MD22 were scheduled on day five of a sleep restriction period and MD42 was day four of a sleep restriction phase.

**Fig. 1:**
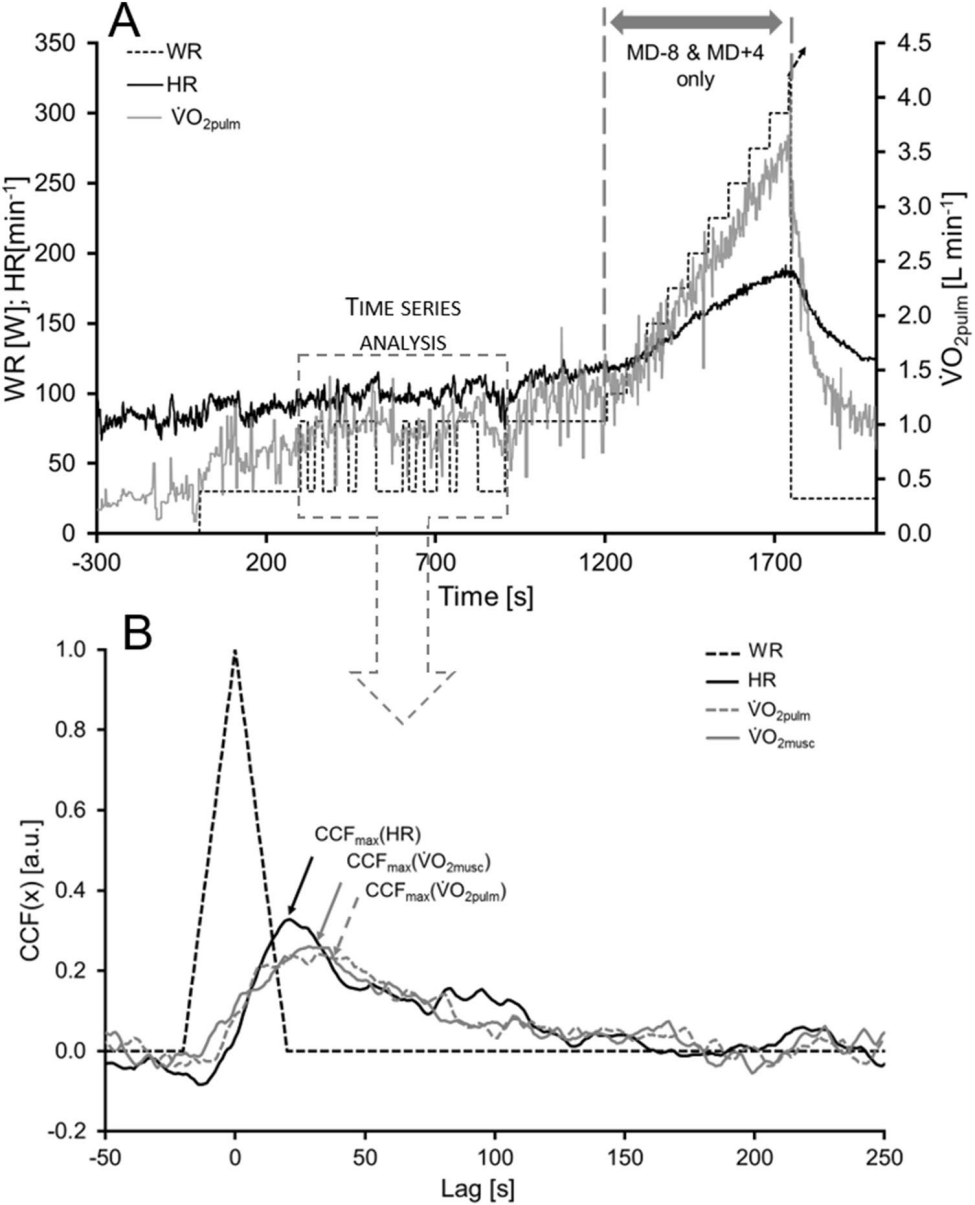
Description of the work rate (WR) protocol (**A**) and the kinetics analysis (B) before, during and after the mission. *HR* heart rate, ${\dot{\text{V}}\text{O}}_{{{\text{2pulm}}}}$ pulmonary oxygen uptake; $\dot{V}O_{2musc}$ muscular oxygen uptake, *MD-8* day 8 before the start of the mission, *MD + 4* four days after the end of the mission, *CCF*_*max*_*(x)* maximum of the cross-correlation function between WR and the respective parameter (x), *CCF(x)* cross-correlation function between WR and the respective parameter (x)

The exercise test was performed on a cycle ergometer (Lode Corival, Lode BV, Groningen, The Netherlands) and consisted of two parts: the first part started with the resting condition, followed by a 300 s phase at a low constant exercise intensity (30 W), which was followed by two PRBS with dynamic WR changes (30 and 80 W) for 300 s each. Thereafter, a constant phase of the higher WR (80 W) was applied for 300 s. The second part of the WR protocol was only performed on MD-8 and MD + 4. On these days, the WR was further increased to 100 W for one minute and afterwards WR was increased by 25 W every minute until fatigue (Fig. 1A).

During all tests, gas exchange was measured breath-by-breath, using a metabolic cart (Metalyzer 3B, Cortex Biophysik GmbH, Leipzig, Germany), including the corrections of Beaver et al. (1981) for alveolar gas exchange. The metabolic cart was calibrated according to the manufacturer’s guidelines, prior to all tests. HR was measured using an ECG (CustoGuard belt 3, CustoMed, Ottobrunn, Germany) and blood pressure was recorded beat-to-beat via Portpares Model 2 (Finapres Medical Systems, Amsterdam, The Netherlands). Using the blood pressure values, stroke volume (SV) was calculated applying the Modelflow Algorithm of the beatscope software (Finapres Medical Systems, Amsterdam, The Netherlands), considering age, height and body mass.

Breath-by-breath data were interpolated step-wise and beat-to-beat data linearly to 1-s-intervals to obtain homogenous sampling.

Peak data of ${\dot{\text{V}}\text{O}}_{{2}}$, HR and WR before and after the mission are reported as averages over the last 30 s before the termination of the WR protocol. The first VT (VT_1_) was determined using the V-slope method in combination with the ventilatory equivalents and endtidal values of O_2_ and CO_2_, as suggested by Beaver et al. (1986).

To receive the kinetics information of the data, time-series analysis was applied. The PRBS signal was auto-correlated (ACF) and interpreted as a WR impulse (Bennett et al. 1981). The Cross-correlation functions (CCFs) between WR and the respective parameters as i.e., HR and pulmonary ${\dot{\text{V}}\text{O}}_{{2}}$ (${\dot{\text{V}}\text{O}}_{{{\text{2pulm}}}}$) were calculated and interpreted as the parameters’ response to this WR impulse. The higher the maximum of the CCF (CCF_max_), the faster was the kinetics response. The greater the lag between the CCF_max_ and the maximum of the ACF (CCF_lag_), the more time delayed was the onset of the response (compare Fig. 1B). Muscular ${\dot{\text{V}}\text{O}}_{{2}}$ (${\dot{\text{V}}\text{O}}_{{{\text{2musc}}}}$) was estimated from HR and $\dot{V}$ O_2pulm_, using the approach of Hoffmann et al. (2013). In this method, a specific venous volume in combination with ${\dot{\text{V}}\text{O}}_{{2}}$ and perfusion of the remainder of the body is estimated to calculate transit times of the ${\dot{\text{V}}\text{O}}_{{{\text{2musc}}}}$ signal from muscle to mouth.

### Statistical analysis

Normal distribution of the respective parameters was tested applying the Kolmogorov–Smirnov test. If normal distribution could be assumed, analysis of variance (ANOVA) with repeated measures on the factor *day* (MD-8, MD22, MD42, MD + 4) and Bonferroni post hoc tests were calculated for the CCF_lag_ and CCF_max_ values. In case normal distribution could not be assumed, Friedman tests with Wilcoxon post hoc tests were used and the False Discovery Rate correction procedure (Benjamini and Hochberg 1995) for multiple testing was applied. ANOVA with the factors *day* (MD-8, MD22, MD42, MD + 4) and *step* [Rest, 30 W, 53.3 W (2xPRBS), 80 W] with repeated measures on both factors were applied to compare the mean values of HR, ${\dot{\text{V}}\text{O}}_{{{\text{2pulm}}}}$, mean arterial blood pressure (mBP), SV and cardiac output (${\dot{\text{Q}}}$). In case sphericity could not be assumed, the Huynh–Feldt correction was used to assess the inner subject effects. Bonferroni post hoc tests were applied, if appropriate. The Pearson product-moment correlation coefficient (one-tailed) was calculated for normally distributed data to test for correlations. The level of significance was set to *α* = 5%. All statistical tests were performed using SPSS 26 (IBM, Amonk, New York, USA).

## Results

The results of the peak exercise test before and after the mission are shown in Table 2.

**Table 2:** Peak values before and after the mission as well as exercise habits before the mission

*N* = 14	MD-8		MD + 4		Sig
	Mean	SD	Mean	SD	
WR_peak_ (W)	257	63	259	56	0.661
HR_peak_ (min^−1^)	180	12	179	12	0.506
$\dot{V}$CO_2peak_ (L min^−1^)	3.45	0.97	2.82	0.62	0.747
$\dot{V}$O_2peak_ (ml kg^−1^ min^−1^)	37.79	5.85	38.92	4.62	0.221
RER_peak_ (a.u.)	1.23	0.07	1.21	0.06	0.486
WR @ VT_1_ (W)	163	34	182	36	0.033
HR @ VT_1_ (min^−1^)	156	19	156	13	0.826
$\dot{V}$O_2_ @VT_1_ (L min^−1^)	1.92	0.35	2.04	0.44	0.135

*MD-8* 8 days before the beginning of the mission, *MD + 4* 4 days after the end of the mission, *VT*_*1*_ first ventilatory threshold, *WR*_*peak*_ peak work rate, *HR*_*peak*_ peak heart rate, $\dot{V}$*CO*_*2peak*_ peak carbon dioxide output, $\dot{V}$*O*_*2peak*_ peak oxygen uptake, *RER*_*peak*_ peak respiratory exchange ratio, *WR @ VT*_*1*_ work rate at the first ventilatory threshold, *HR @ VT*_*1*_ heart rate at the first ventilatory threshold, $\dot{V}$*O*_*2*_* @ VT*_*1*_ oxygen uptake at the first ventilatory threshold

Over the 12 months prior to the start of the mission, the participants exercised 15 ± 6 times per month and about 46 ± 33 min each day.

Comparing the mean values during the different WR steps of the exercise protocol, HR, ${\dot{\text{V}}\text{O}}_{{{\text{2pulm}}}}$, mBP and SV, but not ${\dot{\text{Q}}}$ showed significant differences over time (see Table 3). Only HR showed a significant interaction for time × step (post hoc results are shown in Table 3). Independent of the steps during the WR protocol, ${\dot{\text{V}}\text{O}}_{{{\text{2pulm}}}}$ was significantly higher at MD-8 compared with MD22 (*p* = 0.009) and MD42 (*p* = 0.003). Similarly, mBP values were significantly higher at MD-8 in comparison with MD22 (*p* = 0.028) and MD42 (*p* = 0.007). At MD-8, SV was significantly lower compared with MD42 (*p* = 0.031). Further post hoc results of the interaction effects (time × step) are shown in Table 3.

**Table 3:** Means and standard deviations for the different steps of the work rate protocol

*N* = 14	Phase	MD-8			MD22			MD42			MD + 4			Time	Step	Time × step
		Mean	SD		Mean	SD		Mean	SD		Mean	SD				
HR (min^−1^)	Rest	86	11		80	8		79	14		86	10		< 0.001	< 0.001	0.009
	30	97	13	^a, b^	88	11	^c, d^	87	11	^c, d^	95	11	^a, b^			
	53.3	109	15	^a, b^	97	13	^c, d^	96	11	^c, d^	105	13	^a, b^			
	53.3	113	17	^a, b^	99	14	^c, d^	99	13	^c, d^	109	14	^a, b^			
	80	130	21	^a, b^	111	13	^c, d^	111	13	^c, d^	125	18	^a, b^			
$\dot{V}$O_2pulm_ (L min^−1^)	Rest	0.37	0.08		0.36	0.09		0.33	0.06		0.37	0.08		< 0.001	< 0.001	0.758
	30	0.75	0.08	^b^	0.71	0.07		0.69	0.09	^c^	0.72	0.07				
	53.3	0.95	0.08	^a, b^	0.90	0.06	^c^	0.89	0.06	^c, d^	0.93	0.07	^b^			
	53.3	0.97	0.07	^a, b^	0.92	0.06	^c^	0.90	0.06	^c^	0.94	0.08				
	80	1.25	0.07	^b^	1.14	0.20		1.13	0.17	^c^	1.21	0.11				
mBP (mmHg)	Rest	95	14		89	12		87	8		91	9		0.001	< 0.001	0.087
	30	105	12	^a, b^	90	12	^c^	89	6	^c^	94	10				
	53.3	105	12	^a, b^	93	12	^c^	92	7	^c^	96	10				
	53.3	104	11	^a, b^	92	10	^c^	90	8	^c^	95	9				
	80	105	13	^a, b^	93	11	^c^	93	9	^c, d^	99	9	^b^			
SV (ml)	Rest	68	19		68	14		73	19		75	15		0.045	< 0.001	0.227
	30	82	31		96	26		97	34		92	18				
	53.3	87	32	^b^	99	24		108	35	^c^	97	21				
	53.3	91	30		102	25		105	28		96	19				
	80	94	31		99	27		102	35		101	19				
$\dot{Q}$ (L min^−1^)	Rest	5.83	1.53		5.39	1.29		5.79	2.12		6.27	1.41		0.652	< 0.001	0.296
	30	7.89	2.85		8.44	2.42		8.35	3.00		8.26	2.88				
	53.3	9.31	2.88		9.55	2.62		10.25	3.16		9.91	2.80				
	53.3	10.02	2.73		9.96	2.46		10.23	2.54		10.37	2.36				
	80	11.97	3.35		10.91	3.15		11.10	3.37		12.36	2.54				

*Phase* phase of the test protocol, *WR* work rate, *HR* heart rate, $\dot{V}$O_2pulm_ pulmonary oxygen uptake, *mBP* mean arterial pressure, *SV* stroke volume, $\dot{Q}$ cardiac output, *MD-8* 8 days before the beginning of the isolation period, *MD22* Day 22 of the isolation period, *MD42* Day 42 of the isolation period, *MD + 4* 4 days after end of the isolation period

^a^Significantly different to MD22

^b^Significantly different to MD42

^c^Significantly different to MD-8

^d^Significantly different to MD + 4

Comparing the CCF_max_ and CCF_lag_ values of ${\dot{\text{V}}\text{O}}_{{{\text{2musc}}}}$, HR, ${\dot{\text{V}}\text{O}}_{{{\text{2pulm}}}}$ and mBP between the different mission days, significant differences were observed for CCF_max_(HR) only (*p* = 0.008). Post hoc results are shown in Table 4.

**Table 4:** Kinetics responses during the exercise test

*N* = 14		MD-8		MD22		MD42		MD + 4		Sig. for factor time
		Mean	SD	Mean	SD	Mean	SD	Mean	SD	
$\dot{V}$O_2musc_	CCF_max_ [a.u.]	0.35	0.05	0.35	0.05	0.38	0.06	0.35	0.05	0.354
	CCF_lag_ [s]	22	10	26	9	22	10	25	14	0.770
HR	CCF_max_ [a.u.]	0.30^b,c,d^	0.06	0.36^a^	0.06	0.38^a^	0.06	0.35^a^	0.06	0.008
	CCF_lag_ [s]	24	29	19	6	26	21	21	18	0.142
$\dot{V}$O_2pulm_	CCF_max_ [a.u.]	0.35	0.06	0.35	0.06	0.35	0.04	0.34	0.05	0.781
	CCF_lag_ [s]	31	14	33	7	30	11	36	15	0.326
mBP	CCF_max_ [a.u.]	0.25	0.07	0.28	0.09	0.26	0.05	0.23	0.04	0.216
	CCF_lag_ [s]	108	73	116	94	73	77	88	86	0.653

$\dot{V}$O_2musc_ muscular oxygen uptake, *HR* heart rate, $\dot{V}$O_2pulm_ pulmonary oxygen uptake, *mBP* mean arterial pressure, *MD-8* 8 days before the beginning of the isolation period, *MD22* Day 22 of the isolation period, *MD42* Day 42 of the isolation period, *MD + 4* 4 days after the end of the mission, *Sig* significant effect

^a^Significantly different to MD-8 (*p* ≤ 0.05)

^b^Significantly different to MD22 (*p* ≤ 0.05)

^c^Significantly different to MD42 (*p* ≤ 0.05)

^d^Significantly different to MD + 4 (*p* ≤ 0.05)

**Fig. 2:**
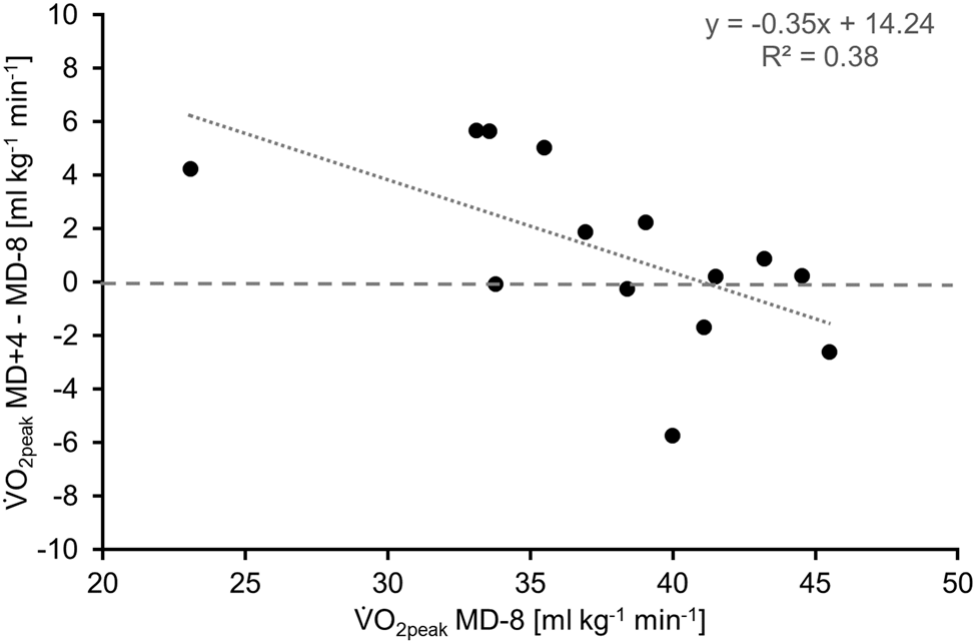
Correlation (*r* = − 0.614, *p* = 0.010) between peak oxygen uptake (${\dot{\text{V}}\text{O}}_{{{\text{2peak}}}}$)on MD-8 and the difference in ${\dot{\text{V}}\text{O}}_{{{\text{2peak}}}}$ from pre to post test. Negative values on the ordinate indicate decreases in ${\dot{\text{V}}\text{O}}_{{{\text{2peak}}}}$ from pre to post test (*N* = 14). MD-8: 8 days before the beginning of the mission, MD + 4: 4 days after the end of the mission

Significant correlations were found between the change in ${\dot{\text{V}}\text{O}}_{{{\text{2peak}}}}$ from MD-8 to MD + 4 and the baseline ${\dot{\text{V}}\text{O}}_{{{\text{2peak}}}}$ values at MD-8 (*r* = − 0.614, *p* = 0.010; Fig. 2).

**Fig. 3:**
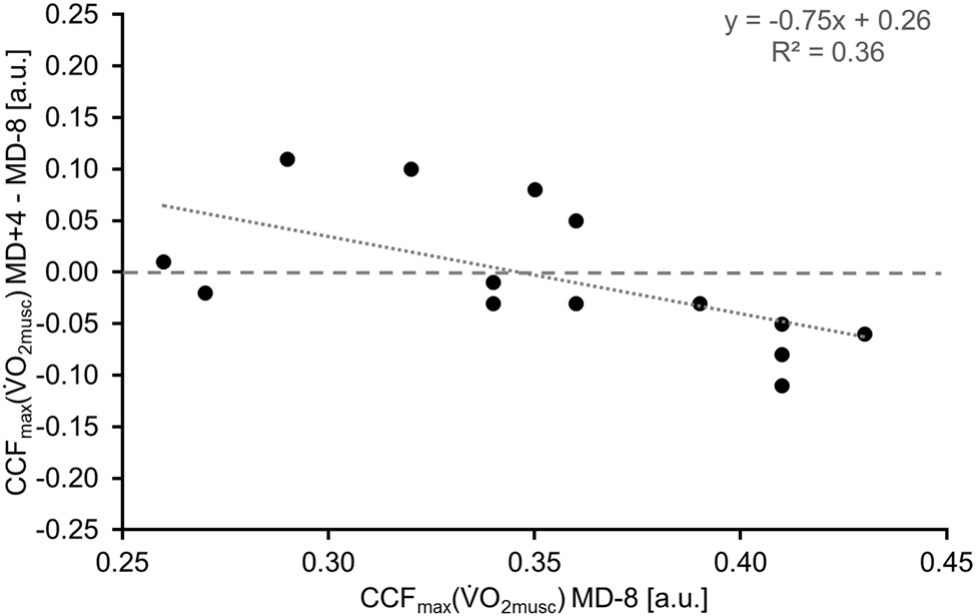
Correlation (*r* = − 0.641, *p* = 0.007) between muscular oxygen uptake kinetics (CCF_max_ (${\dot{\text{V}}\text{O}}_{{{\text{2musc}}}}$)) on MD-8 and the difference in CCF_max_ (${\dot{\text{V}}\text{O}}_{{{\text{2musc}}}}$) from pre to post test. Negative values on the ordinate indicate lower CCF_max_ (${\dot{\text{V}}\text{O}}_{{{\text{2musc}}}}$) from pre to post test (*N* = 14). MD-8: 8 days before the beginning of the mission, MD + 4: 4 days after the end of the mission

Further, the change in CCF_max_(${\dot{\text{V}}\text{O}}_{{{\text{2musc}}}}$) from MD-8 to MD + 4 correlated significantly with the baseline value (MD-8) of CCF_max_(${\dot{\text{V}}\text{O}}_{{{\text{2musc}}}}$) (*r* = − 0.763, *p* = 0.001; Fig. 3).

## Discussion

The aim of the experiment was to show the effect of 45 days of confinement in combination with the prescribed exercise training and sleep restrictions on parameters of exercise capacity and cardiorespiratory regulation. In accordance with the proposed hypothesis, (i) neither ${\dot{\text{V}}\text{O}}_{{{\text{2peak}}}}$ nor ${\dot{\text{V}}\text{O}}_{{{\text{2musc}}}}$ kinetics changed significantly over the different test days during the HERA C4 missions. Therefore, the combination of confinement and sleep restrictions did not affect overall exercise capacity and the regulation of ${\dot{\text{V}}\text{O}}_{{{\text{2musc}}}}$. However, CCF_max_(HR) was higher during and after the mission, when compared with the values before the mission. Hence, HR kinetics can be interpreted as faster during and after the simulated space mission, which is in contrast to the established hypothesis of slower HR kinetics as a result of the combination of partial sleep deprivation and restricted physical activity during the confinement phase (ii). Further, absolute values of HR were higher before and after the mission, compared with the values during the mission.

### Oxygen uptake–peak values and its regulation

The ${\dot{\text{V}}\text{O}}_{{{\text{2peak}}}}$ values did not change significantly from MD-8 to MD + 4. Therefore, the applied exercise program was sufficient to mitigate the potentially deconditioning effects of lower physical activity throughout the 45 d of confinement in combination with sleep restrictions. However, the small but significant increase in WR of 20 W at VT_1_ after the end of the mission (MD + 4), suggests a slightly increased exercise tolerance. Similar results were found for a submarine crew, living in confinement for 70 d. ${\dot{\text{V}}\text{O}}_{{{\text{2peak}}}}$ did not change significantly, but VT_1_ as a percentage of ${\dot{\text{V}}\text{O}}_{{{\text{2peak}}}}$ increased after 8 weeks of endurance exercise training, which is comparable to the effects of the exercise training program applied during HERA C4 (Bennett et al. 1985). However, correlation analyses revealed, that HERA crew members with greater ${\dot{\text{V}}\text{O}}_{{{\text{2peak}}}}$ and faster ${\dot{\text{V}}\text{O}}_{{{\text{2musc}}}}$ kinetics before the mission showed greater declines in the respective parameters after the mission. Moraes et al. (2018), analyzing ${\dot{\text{V}}\text{O}}_{{{\text{2peak}}}}$ values of differently trained crew members (mountaineers and scientists) before and after an Antarctic field expedition reported comparable findings. Similar results were reported for Astronauts during spaceflight (Moore et al. 2014): the ${\dot{\text{V}}\text{O}}_{{{\text{2peak}}}}$ did not decrease in all crew members, but those starting with higher levels of ${\dot{\text{V}}\text{O}}_{{{\text{2peak}}}}$ were more prone to losses at the first inflight test, and those who maintained their preflight ${\dot{\text{V}}\text{O}}_{{{\text{2peak}}}}$ values after return to Earth, spent about 80% of their inflight aerobic training time at ~ 80% of their maximum HR (Moore et al. 2014). For the HERA crew, training prescriptions should have been more customized for the individual training status of the crew members, to apply individually adequate training stimuli throughout the mission phase. Highly trained individuals need a higher training volume to sustain their personal fitness level, which is also known from longitudinal ageing studies (Katzel et al. 2001).

The training effect, indicated by an increased WR at VT_1_ did not result in faster ${\dot{\text{V}}\text{O}}_{{{\text{2musc}}}}$ kinetics. This suggests different timelines for the adjustment of peak exercise capacities (${\dot{\text{V}}\text{O}}_{{{\text{2peak}}}}$), submaximal aerobic capacities (VT_1_) and regulatory parameters (kinetics) at muscular level.

### Heart rate–mean values and regulatory aspects

In comparison with the pre and post-tests, mean HR values during the constant phases of the WR protocol during the mission phase were lower. However, HR kinetics at MD-8 were slower compared with MD22, MD42 and MD + 4. Hence, changes in the absolute HR values during the WR step and the speed of adjustment of HR (kinetics) in response to increased metabolic demands are not similar. Regarding the altered regulatory behavior of HR, a training effect of the exercise countermeasure can be concluded, which is not evident in the mean values of HR. Therefore, two aspects should be considered: (1) a training effect via the exercise countermeasure and (2) autonomic system changes caused by reduced external stimuli. Regarding the effect of the exercise countermeasure (1), the accelerated HR kinetics starting at MD22 are in accordance with the increased WR at VT1 after the end of the mission (MD + 4). For comparison, after bed rest, HR kinetics were significantly slowed as a result of deconditioning (Koschate et al. 2018) and were correlated with changes in ${\dot{\text{V}}\text{O}}_{{{\text{2peak}}}}$ in astronauts before and after their mission to the International Space Station (Hoffmann et al. 2016). After 12 weeks of endurance exercise training, HR kinetics were accelerated in a group of male type 2 diabetes patients (Koschate et al. 2016a). Hence, HR kinetics seem to be a sensitive parameter to observe changes in cardiovascular regulation in response to exercise training stimuli. These training effects were not visible in the mean values of HR during the exercise test throughout the HERA missions. Considering the potential changes in the autonomic nervous system due to the confinement phase (2), either a higher parasympathetic system activity during the mission or an increased sympathetic system activity before and after the mission (Coote 2010) should be considered, which might (although not statistically significant between MD42 and MD + 4) also slightly dampen the potential training effect, which was observed for HR kinetics at MD42. During Russian confinement experiments with durations of 120 and 520 d (Vigo et al. 2012, 2013), a greater HRV and reduced cortical activation levels were reported (Weber et al. 2020; Jacubowski et al. 2015). Greater HRV was suggested to be influenced by boredom, artificial light or greater parasympathetic system activity during isolation (Vigo et al. 2012, 2013). These circumstances might have altered the exercise HR during the HERA C4 missions as well.

In experiments, applying acute and chronic (partial) sleep deprivation, reduced HR variability, higher HR, increases in sympathovagal activity and decreases in vagal activity in the resting condition and/or during sleep were described (Tobaldini et al. 2017a, b; Dettoni et al. 2012; Glos et al. 2014; Fogt et al. 2011; Liu et al. 2015). Dettoni et al. (2012) found increased sympathetic activity in the resting supine position after only five nights of partial sleep restriction, which is comparable to the HERA campaign (5 h). Using linear mixed-effects models, significant linear relationships were found between fatigue level and HRV (Fogt et al. 2011). This is in contrast to the observed cardiovascular data during exercise in this experiment. The findings of the present experiment, supported by the data of Vigo et al. (2012, 2013) suggest, that the effect of confinement and reduced external stimuli in combination with the applied exercise countermeasure during the simulated mission to an asteroid might have had a greater effect on the cardiovascular system, than partial sleep deprivation. Therefore, partial sleep deprivation, at least when combined with the applied exercise countermeasure does not yield negative effects regarding the regulation of the cardiovascular system at moderate WR intensities. In line with this, no correlations were found between baseline ${\dot{\text{V}}\text{O}}_{{{\text{2peak}}}}$, ${\dot{\text{V}}\text{O}}_{{{\text{2musc}}}}$ or pre to post changes in these parameters with HR kinetics.

### Limitations

No further parameters to explain the lower HRs during the mission compared with the pre and post mission values were assessed. Therefore, it remains unclear whether the sympathetic nervous system activity was increased before or decreased during the mission and vice versa, whether the parasympathetic system activity was higher during or lower before and after the mission. The effect of confinement on cardiorespiratory fitness, cannot be evaluated separately from the effect of sleep restrictions, since no test during the mission was performed after recovery sleep.

Two participants had to terminate the test early, because they exceeded threshold values for HR or blood pressure, which were set by the NASA medical board. Hence, for future confinement experiments, a further reduction of the applied WR intensities should be considered.

## Conclusions

The kinetics of HR were significantly faster during and at the end of the simulated space mission including sleep deprivation, compared to the values before the mission. Therefore, HR kinetics seem to indicate changes in cardiovascular regulation very sensitively. Absolute values of HR during the exercise test followed a different timeline, with higher values before and after the mission. Changes in ${\dot{\text{V}}\text{O}}_{{{\text{2musc}}}}$ kinetics and ${\dot{\text{V}}\text{O}}_{{{\text{2peak}}}}$ were not significant, but less fit individuals seemed to benefit more from the applied exercise training countermeasure, than crew members with a high baseline fitness level. Hence, for future missions, the exercise countermeasure protocol should be intensified for people with a higher fitness status. The applied exercise test seems promising for the evaluation of the cardiovascular and respiratory system during circumstances where medical support is not consistently available, or in patients where maximal exercise intensities are not suitable, since it requires only moderate WR intensities and only a short amount of time. Especially, HR kinetics should be investigated in more detail. The kinetics of HR are simple to assess during exercise and provide more sophisticated information about the status of the cardiovascular system, than absolute values of HR alone.

## Data Availability

Data available upon request due to privacy/ethical restrictions.

## Abbreviations

ACF: Auto-correlation function
ANOVA: Analysis of variance
CCFs: Cross-correlation function
CCF_lag_(x): Lag between the maximum of the ACF and the CCF
CCF_max_(x): Maximum of the cross-correlation function
HERA: Human exploration research analog
HR: Heart rate
HRV: Heart rate variability
mBP: Mean blood pressure
MD: Mission day
NASA: National aeronautics and space administration
PRBS: Pseudo random binary sequences
$${\dot{\text{Q}}}$$: Cardiac output
SV: Stroke volume
$${\dot{\text{V}}\text{O}}_{{2}}$$: Oxygen uptake
${\dot{\text{V}}\text{O}}_{{{\text{2musc}}}}$: Muscular oxygen uptake
${\dot{\text{V}}\text{O}}_{{{\text{2peak}}}}$: Peak oxygen uptake
${\dot{\text{V}}\text{O}}_{{{\text{2pulm}}}}$: Pulmonary oxygen uptake
VT: Ventilatory threshold
VT_1_: First ventilatory threshold
WR: Work rate
